# Independent Risk Factors for Injury in Pre-School Children: Three Population-Based Nested Case-Control Studies Using Routine Primary Care Data

**DOI:** 10.1371/journal.pone.0035193

**Published:** 2012-04-05

**Authors:** Elizabeth Orton, Denise Kendrick, Joe West, Laila J. Tata

**Affiliations:** 1 Division of Primary Care, University Park, University of Nottingham, Nottingham, United Kingdom; 2 Division of Epidemiology and Public Health, Nottingham City Hospital, University of Nottingham, Nottingham, United Kingdom; The University of Hong Kong, Hong Kong

## Abstract

**Background:**

Injuries in childhood are largely preventable yet an estimated 2,400 children die every day because of injury and violence. Despite this, the factors that contribute to injury occurrence have not been quantified at the population scale using primary care data. We used The Health Improvement Network (THIN) database to identify risk factors for thermal injury, fractures and poisoning in pre-school children in order to inform the optimal delivery of preventative strategies.

**Methods:**

We used a matched, nested case-control study design. Cases were children under 5 with a first medically recorded injury, comprising 3,649 thermal injury cases, 4,050 fracture cases and 2,193 poisoning cases, matched on general practice to 94,620 control children.

**Results:**

Younger maternal age and higher birth order increased the odds of all injuries. Children’s age of highest injury risk varied by injury type; compared with children under 1 year, thermal injuries were highest in those age 1-2 (OR = 2.43, 95%CI 2.23–2.65), poisonings in those age 2-3 (OR = 7.32, 95%CI 6.26–8.58) and fractures in those age 3-5 (OR = 3.80, 95%CI 3.42–4.23). Increasing deprivation was an important modifiable risk factor for poisonings and thermal injuries (tests for trend p≤0.001) as were hazardous/harmful alcohol consumption by a household adult (OR = 1.73, 95%CI 1.26–2.38 and OR = 1.39, 95%CI 1.07–1.81 respectively) and maternal diagnosis of depression (OR = 1.45, 95%CI 1.24–1.70 and OR = 1.16, 95%CI 1.02–1.32 respectively). Fracture was not associated with these factors, however, not living in single-adult household reduced the odds of fracture (OR = 0.88, 95%CI 0.82–0.95).

**Conclusions:**

Maternal depression, hazardous/harmful adult alcohol consumption and socioeconomic deprivation represent important modifiable risk factors for thermal injury and poisoning but not fractures in preschool children. Since these risk factors can be ascertained from routine primary care records, pre-school children’s frequent visits to primary care present an opportunity to reduce injury risk by implementing effective preventative interventions from existing national guidelines.

## Introduction

Childhood injury is largely preventable [1] yet continues to be a significant public health issue. Globally an estimated 2,400 children die every day due to injury and violence [2] and many more are disabled or require substantial medical intervention. In high income countries injuries still account for 40% of all child deaths between age 1-14 [3] and in the United Kingdom (UK) injuries rank among the leading causes of death in children aged 1-4 years [4]. The most common injuries in this age group are falls, poisonings and thermal injuries which result in over a quarter of a million emergency department attendances [5] costing more than £17 million [6] and resulting in more than 20,000 hospital admissions each year [7].

Recent guidance in the UK from the National Institute for Health and Clinical Excellence (NICE) recommends identifying those children at greatest risk of injury in order to target the provision of home safety interventions [8]. Interventions include conducting home safety assessments and installing safety equipment as deemed necessary. NICE also suggest that GPs along with other primary care practitioners such as Health Visitors should refer families that would benefit from these interventions and that GPs and other primary care practitioners should provide home safety advice when visiting the home of vulnerable families, even if this is not related to a safety or injury issue. However there is little evidence of a strategic or comprehensive response to the problem of childhood injury within the National Health Service (NHS) [6]. For the implementation of preventative measures to be effective, we require both a comprehensive understanding of the common factors that put children at risk of avoidable injury and methods for identifying these children in order to have impact at a population level, however both of these are currently lacking.

Previous studies have identified that individual [9]-[17], family [11]–[28] and community level factors [9]-[15], [17], [29]–[32] contribute to children’s risk of injury yet the actual contributions of these factors to the burden of children’s injuries in the general population remain unclear. Most studies were conducted in small geographic areas [9], [11], [13], [15], [19], [22], [31] and used only injuries reported to secondary care [9], [10], [12], [13], [15], [16], [18], [20], [23], [30]–[32] which represent a highly select group of injuries at the severe end of the spectrum. In addition, common use of parental reporting of both risk factors and injuries [11], [14], [19]–[26] combined with cross-sectional designs [14], [21]–[23], [25], [31], [33] will have introduced social desirability and recall biases. Most importantly, there has been a lack of specific injury definitions, potentially masking important differences in risk factors between injury types or mechanisms.

We have therefore undertaken a study to quantify the effects of risk factors on the first occurrence of common childhood injuries using a nationally-representative dataset as no large scale studies have addressed this issue previously in the UK. If primary care data can be used in this way it will assist compliance with NICE guidance recommendations that interventions are implemented in primary care by offering home safety assessments and equipment schemes to those at greatest risk and addressing inequities in child injury[8].

## Methods

### Ethics Statement

We used The Health Improvement Network (THIN) primary care data for this research. The company that own THIN (Cegedim Strategic Data Medical Research) has received ethical approval for studies using only pre-collected, anonymised data to undergo only a scientific review. This applies to our study and we have complied fully with this procedure. A research protocol was submitted to the Scientific Review Committee and the protocol was approved in October 2009. Patient informed consent is not required under this agreement nor is additional ethics approvals from either the National Health Service ethics committees or from The University of Nottingham.

### Study Design, Setting and Participants

We used prospectively collected longitudinal data from The Health Improvement Network (THIN), a computerised primary care database of anonymised patient records from general practices across the UK. GPs are notified of all healthcare provision for each patient, including treatment in secondary and tertiary care which should be entered into the electronic record. Practices that contribute are broadly representative of all general practices in the UK in terms of age and sex of patients, practice size, geographical distribution and data quality [34]. At the time of data extraction, THIN comprised 255 general practices across England, Wales, Scotland and Northern Ireland with 3.9 million patient records.

The study population consisted of an open cohort of all children in THIN born between January 1988 and November 2004 who were linked to their mothers’ general practice records as described previously [35]. All children were registered with their general practice within 60 days of birth to ensure identification of the first occurrence of injury and risk factors from birth onwards. One child was then selected at random from each household to avoid clustering of common risk factors for multiple children in the same household. Other household members were identified using a household code within THIN.

From this birth cohort we identified all first occurrences of thermal injury, fracture and poisoning up to the age of 5 years and created three respective case-control datasets to assess risk factors associated with each specific type of injury. For each dataset, cases were defined as children who had a medical code specific to the injury type in their record. If a case had a repeated injury of the same type (e.g. medical codes for fracture at age 2 and again at age 4 years), they were defined by only their first injury. For each case we matched up to 10 controls who were children under age 5 years, registered with the same general practice as the case at the date of the case’s injury and did not have a medical code for the case’s type of injury up to that date. To ensure that cases and controls in each study were representative of the whole birth cohort, controls in one study were eligible to be cases in one of the other case-control studies and vice versa.

### Risk Factor Variables

We assessed child, maternal and household risk factors available in THIN that had been identified in previous studies as potential independent risk factors for childhood injury [9]-[32], [36]–[38]. Child covariates included sex of the child, age at the time of the case’s injury and birth order. Maternal covariates included the mother’s age when the child was born and perinatal depression defined as a diagnosis of depression during pregnancy or in the first 6 months after delivery.

By identifying general practice records of other individuals in the same household we obtained data on the following household covariates: socioeconomic status using quintiles of the Townsend Index of material deprivation [39] which are linked to each patient’s home postcode (the Townsend index is a composite score comprising measures of employment, car ownership, home ownership and overcrowding), household composition (i.e. number of adults) and hazardous or harmful alcohol consumption by any adult in the household during the lifetime of the child, but recorded prior to the case’s injury. Household composition was estimated by counting the number of adults in the household over 16 years of age. Hazardous or harmful alcohol consumption was defined using medical codes for severe morbidity (e.g. alcohol withdrawal syndrome, alcohol dependence syndrome), problematic drinking (e.g. alcohol problem drinking, nondependent alcohol abuse), specific treatment for alcohol addiction (e.g. alcohol detoxification, under care of community alcohol team) or specific reference to frequent high levels of consumption (e.g. heavy drinker–7–9 u/day).

### Statistical Methods

Characteristics of cases and controls were described using frequencies and percentages. Conditional logistic regression was used to estimate bivariable and multivariable odds ratios (OR) with 95% confidence intervals (CI) for each injury type associated with potential risk factors at the child, maternal and household levels. Multivariable models were built using the procedure described by Collett and assessing significance using likelihood ratio tests (LRT) [40]. All covariates described above were included in the initial model and child age and sex were retained as *a priori* confounders. Potential interactions between covariates were identified *a priori* based on theoretical plausibility. For example, we tested interactions between birth order and age at the time of injury and also the age of the child at the time of injury and the age of the mother when the child was born. Interaction terms were added to the model, significance assessed using a LRT and those with a *p*-value of <0.01 (in view of the large sample size) were retained in the model. The final model was tested for multicollinearity using the covariate correlation matrix and by calculating the variance inflation factor. Missing data were included as a separate category in the analysis.

We estimated the statistical power of our analyses using the rarest risk factor in each of the 3 case-control studies, hazardous or harmful alcohol consumption in the household, which had a prevalence of 1.3% in the controls. To obtain 80% power to detect an odds ratio of 1.65 at the 5% significance level, assuming a correlation for exposures between cases and controls of 0.2 to allow for matching on general practice, we required 2,174 cases and 10 matched controls for each study. For all other exposures, we had much greater statistical power.

For modifiable risk factors (perinatal depression, hazardous or harmful alcohol consumption and socioeconomic deprivation) we estimated the proportion of each injury type that could potentially be averted if the risk factor was not present by calculating attributable risk fractions amongst the exposed cases [41].

All analyses were conducted using Stata version SE11.

## Results

The characteristics of cases and controls are presented in Table 1. The study population consisted of an open cohort of 180,064 linked mother/child pairs from which 3,649 first occurrences of thermal injury, 4,050 first occurrences of fracture and 2,193 first occurrences of poisoning were identified from children’s records. For these three case groups, 34,395, 38,852, 21,373 controls were selected for thermal injuries, fractures and poisonings respectively. Distributions of risk factors were the same across the three control groups showing that they represented the same baseline population. Bivariable associations between risk factors and specific injury type are shown in Table 2 and the fully adjusted multivariable models for each injury type are shown in Table 3.

**Table 1:** Characteristics of cases with specific injury types and their respective controls from the general population.

		Thermal injuries		Fractures		Poisoning	
		Case N = 3,649 Frequency (%)	Control N = 34,395 Frequency (%)	Case N = 4,050 Frequency (%)	Control N = 38,852 Frequency (%)	Case N = 2,193 Frequency (%)	Control N = 21,373 Frequency (%)
**Sex**	Female	1,529 (41.9)	16,831 (48.9)	1,890 (46.7)	18,987 (48.9)	1,036 (47.2)	10,487 (49.1)
	Male	2,120 (58.1)	17,564 (51.1)	2,160 (53.3)	19,865 (51.1)	1,157 (52.8)	10,886 (50.9)
**Age of child at injury**	0-12 months	1,030 (28.2)	12,118 (35.2)	531 (13.1)	12,928 (33.3)	221 (10.1)	7,272 (34.0)
	13-24 months	1,536 (42.1)	7,555 (22.0)	1,009 (24.9)	8,319 (21.4)	798 (36.4)	4,680 (21.9)
	25-36 months	609 (16.7)	5,779 (16.8)	983 (24.3)	6,825 (17.6)	786 (35.8)	3,826 (17.9)
	37+months	474 (13.0)	8,943 (26.0)	1,527 (37.7)	10,780 (27.7)	388 (17.7)	5,595 (26.2)
**Birth order**	1st child	2,351 (64.4)	24,642 (71.6)	2,444 (60.3)	27,510 (70.8)	1,422 (64.8)	15,221 (71.2)
	2nd child	1,049 (28.8)	8,344 (24.3)	1,319 (32.6)	9,668 (24.9)	629 (28.7)	5,236 (24.5)
	3rd or later	249 (6.8)	1,409 (4.1)	287 (7.1)	1,674 (4.3)	142 (6.5)	916 (4.3)
**Age of mother at birth of child**	Under 20	341 (9.3)	1,898 (5.5)	226 (5.5)	2,235 (5.7)	194 (8.9)	1,194 (5.6)
	20-29	1,904 (52.2)	16,801 (48.8)	2,065 (51.0)	18,877 (48.6)	1,211 (55.2)	10,562 (49.4)
	30-39	1,333 (36.5)	14,889 (43.3)	1,679 (41.5)	16,845 (43.4)	744 (33.9)	9,098 (42.6)
	40 and over	71 (2.0)	807 (2.4)	80 (2.0)	895 (2.3)	44 (2.0)	519 (2.4)
**Perinatal depression**	No	3,344 (91.6)	31,986 (93.0)	3,767 (93.0)	36,290 (93.4)	1,969 (89.8)	19,861 (92.9)
	Yes	305 (8.4)	2,409 (7.0)	283 (7.0)	2,562 (6.6)	224 (10.2)	1,512 (7.1)
**Hazardous/harmful alcohol consumption in household**	No	3,578 (98.1)	33,938 (98.7)	3,988 (98.5)	38,347 (98.7)	2,142 (97.7)	21,086 (98.7)
	Yes	71 (1.9)	457 (1.3)	62 (1.5)	505 (1.3)	51 (2.3)	287 (1.3)
**Household composition**	Single adult	1,570 (43.0)	13,438 (39.1)	1,686 (41.6)	15,160 (39.0)	945 (43.1)	8,294 (38.8)
	Two adult	1,723 (47.2)	17.713 (51.5)	2,040 (50.4)	20,104 (51.8)	1,069 (48.7)	11,188 (52.4)
	Other family structure	356 (9.8)	3,244 (9.4)	324 (8.0)	3,588 (9.2)	179 (8.2)	1,891 (8.8)
**Deprivation (Townsend Score)**	(1) least deprived	616 (16.9)	7,464 (21.7)	928 (22.9)	9,183 (23.6)	418 (19.1)	4,725 (22.1)
	(2)	549 (15.0)	6,032 (17.5)	732 (18.1)	7,186 (18.5)	335 (15.3)	3,950 (18.5)
	(3)	690 (18.9)	6,255 (18.2)	741 (18.3	7,134 (18.4)	437 (19.9)	4,083 (19.1)
	(4)	762 (20.9)	6,271 (18.3)	748 (18.5)	6,857 (17.6)	443 (20.2)	3,919 (18.3)
	(5) Most deprived	745 (20.4)	5,514 (16.0)	636 (15.7)	5,848 (15.1)	415 (18.9)	3,167 (14.8)
	Not known	287 (7.9)	2,859 (8.3)	265 (6.5)	2,644 (6.8)	145 (6.6)	1,529 (7.2)

**Table 2:** Unadjusted odds ratios for individual injury types and potential risk factors.

		Thermal injuries	Fractures	Poisoning
		Unadjusted Odds Ratios (95% Confidence Interval)	Unadjusted Odds Ratios (95% Confidence Interval)	Unadjusted Odds Ratios (95% Confidence Interval)
**Sex**	Female	1.00	1.00	1.00
	Male	1.33 (1.24-1.42)	1.09 (1.03-1.17)	1.07 (0.98-1.17)
**Age of child at injury**	0-12 months	1.00	1.00	1.00
	13-24 months	2.41 (2.22-2.63)	3.04 (2.72-3.39)	5.78 (4.95-6.75)
	25-36 months	1.24 (1.11-1.38)	3.72 (3.32-4.16)	7.06 (6.04-8.26)
	37+months	0.61 (0.54-0.68)	3.70 (3.33-4.11)	2.36 (1.99-2.81)
**Birth order**	1st child	1.00	1.00	1.00
	2nd child	1.38 (1.27-1.49)	1.59 (1.47-1.71)	1.33 (1.20-1.47)
	3rd or later	2.01 (1.74-2.33)	2.04 (1.78-2.34)	1.76 (1.45-2.13)
**Age of mother at birth of child**	Under 20	1.00	1.00	1.00
	20-29	0.62 (0.55-0.71)	1.10 (0.95-1.27)	0.70 (0.59-0.82)
	30-39	0.49 (0.43-0.56)	1.01 (0.87-1.17)	0.49 (0.41-0.58)
	40 and over	0.48 (0.37-0.63)	0.90 (0.68-1.17)	0.50 (0.36-0.71)
**Perintal depression**	No	1.00	1.00	1.00
	Yes	1.22 (1.08-1.39)	1.06 (0.93-1.20)	1.50 (1.29-1.75)
**Household composition**	Single adult	1.00	1.00	1.00
	Two adult	0.84 (0.78-0.90)	0.92 (0.86-0.99)	0.83 (0.75-0.91)
	Other family structure	0.97 (0.85-1.10)	0.82 (0.72-0.93)	0.82 (0.69-0.98)
**Hazardous/harmful alcohol consumption in household**	No	1.00	1.00	1.00
	Yes	1.49 (1.16-1.93)	1.17 (0.90-1.53)	1.76 (1.30-2.38)
**Townsend Score**	(1) least deprived	1.00	1.00	1.00
	(2)	1.13 (1.00-1.28)	1.01 (0.91-1.12)	0.97 (0.83-1.13)
	(3)	1.44 (1.27-1.62)	1.03 (0.93-1.15)	1.27 (1.09-1.47)
	(4)	1.63 (1.44-1.84)	1.08 (0.97-1.21)	1.38 (1.19-1.61)
	(5) Most deprived	1.85 (1.63-2.11)	1.05 (0.94-1.2)	1.67 (1.42-1.96)
	Not known	1.20 (0.95-1.51)	0.90 (0.71-1.14)	0.99-0.72-1.35)

**Table 3:** Fully adjusted risk factor models for individual injury types.

		Thermal injuries	Fractures	Poisoning
		Adjusted Odds Ratios (95% Confidence Interval)	Adjusted Odds Ratios (95% Confidence Interval)	Adjusted Odds Ratios (95% Confidence Interval)
**Sex**	Female	1.00	1.00	1.00
	Male	1.32 (1.23-1.42)	1.09 (1.02-1.17)	1.07 (0.98-1.18)
**Age of child at injury**	0-12 months	1.00	1.00	1.00
	13-24 months	2.43 (2.23-2.65)	3.02 (2.70-3.37)	5.87 (5.02-6.85)
	25-36 months	1.27 (1.14-1.41)	3.73 (3.33-4.17)	7.32 (6.26-8.58)
	37+months	0.64 (0.57-0.71)	3.80 (3.42-4.23)	2.51 (2.11-2.98)
**Birth order**	1st child	1.00	1.00	1.00
	2nd child	1.43 (1.32-1.55)	1.64 (1.52-1.77)	1.43 (1.28-1.59)
	3rd or later	2.06 (1.77-2.40)	2.17 (1.89-2.49)	1.94 (1.58-2.37)
**Age of mother at birth of child**	Under 20	1.00	1.00	1.00
	20-29	0.61 (0.54-0.70)	0.89 (0.77-1.04)	0.66 (0.56-0.79)
	30-39	0.50 (0.43-0.58)	0.81 (0.69-0.94)	0.48 (0.40-0.58)
	40 and over	0.48 (0.37-0.64)	0.75 (0.57-0.99)	0.46 (0.32-0.67)
**Perinatal depression**	No	1.00	-	1.00
	Yes	1.16 (1.02-1.32)	-	1.45 (1.24-1.70)
**Household composition**	Single adult	1.00	1.00	-
	Two adult	0.90 (0.83-0.97)	0.88 (0.82-0.95)	-
	Other family structure	0.94 (0.82-1.07)	0.89 (0.78-1.01)	-
**Hazardous/ harmful alcohol consumption in household**	No	1.00	-	1.00
	Yes	1.39 (1.07-1.81)	-	1.73 (1.26-2.38)
**Townsend Score**	(1) least deprived	1.00	-	1.00
	(2)	1.11 (0.98-1.26)	-	0.96 (0.82-1.13)
	(3)	1.35 (1.19-1.53)	-	1.23 (1.05-1.43)
	(4)	1.47 (0.30-1.67)	-	1.26 (1.08-1.48)
	(5) Most deprived	1.56 (1.36-1.78)	-	1.48 (1.25-1.75)
	Not known	1.09 (0.86-1.38)	-	0.97 (0.70-1.35)

### Thermal Injuries

Multivariable analysis of thermal injuries showed that male sex (OR 1.32, 95% CI 1.23–1.42) and increasing birth order (test for trend p<0.001) were both associated with increased odds of injury and there was an n-shaped relationship with child age, with the highest odds of injury occurring at age 1-2 years (OR, 2.43 95% CI 2.23–2.65) compared with children under 1 year (Table 3). The odds of thermal injury decreased with increasing maternal age (test for trend p<0.001) and increased if the mother had a diagnosis of depression in the perinatal period (OR 1.16, 95% CI 1.02–1.32). Children living in 2-adult households had a lower odds of injury (OR 0.90, 95% CI 0.83–0.97) compared with those in single adult households. The largest modifiable risk factors for thermal injuries were adult hazardous or harmful alcohol consumption (OR 1.39, 95% CI 1.07–1.81) and increasing socioeconomic deprivation (test for trend p<0.001). No significant interactions were found between covariates.

We estimated that 17% of thermal injuries in children whose mothers suffered from perinatal depression was attributable to depression, 32% of thermal injuries in children in households with hazardous or harmful alcohol consumption was attributable to this alcohol use and 39% of thermal injuries amongst children living in the most deprived quintile of the population was attributable to socioeconomic deprivation.

### Fractures

Multivariable modelling showed a different risk profile for fracture injury compared with thermal injury (Table 3). Male sex (OR 1.09, 95% CI 1.02–1.17), and increasing birth order (test for trend p<0.001) were again associated with increased odds of injury but the odds increased with age, with the highest odds occurring in the 3-5 year old age group (OR 3.80, 95% CI 3.42–4.23) compared with the youngest. The odds of fracture decreased with increasing age of the mother (test for trend p = 0.003) and in households with 2 adults (OR 0.88, 95% CI 0.82–0.95) compared with single adult households. No significant interactions were found between covariates.

Odds ratios for maternal perinatal depression, hazardous or harmful alcohol consumption in the household and socioeconomic deprivation in bivariate analyses were all close to unity (Table 2) and none of these modifiable risk factors were retained in the final multivariable model for fractures.

### Poisonings

The risk factor profile for poisonings was most similar to that for thermal injuries in the fully adjusted model. Although male sex was not associated with poisonings (OR 1.07, 95% CI 0.98–1.18), odds were higher with increasing birth order (test for trend p<0.001) and younger maternal age (test for trend p<0.001) and there was an even steeper n-shaped relationship with child age, with the highest odds of injury occurring at age 2–3 years (OR 7.32, 95% CI 6.26–8.58) compared with those under 1 year. Increasing odds ratios for poisonings associated with each quintile of socioeconomic deprivation were very similar to those for thermal injuries (test for trend p = 0.001). Having a mother with perinatal depression or living in a household in which an adult that had hazardous or harmful alcohol consumption were even stronger independent risk factors for poisonings (OR 1.45, 95% CI 1.24–1.70 and OR 1.73, 95% CI 1.26–2.38 respectively). No significant interactions were found between covariates.

Hazardous or harmful alcohol consumption conferred the highest attributable risk for poisonings with 43% of these injuries in children living in households with hazardous or harmful alcohol consumption being attributable to this alcohol use. The equivalent attributable risk fractions for children whose mothers’ had perinatal depression and those living in the most socioeconomically deprived quintile of the population were 33% and 32% respectively.

## Discussion

Information routinely available in primary care records was effective in identifying important differences in risk profiles for children’s thermal injuries, fractures and poisonings. In particular we identified maternal depression, hazardous or harmful adult alcohol use and socioeconomic deprivation as important modifiable factors in a child’s risk of both thermal and poisonings but not fracture. For example, children’s odds of thermal injury were 40% higher and 70% higher for poisonings if there was an adult in the household with a record of hazardous or harmful alcohol consumption and, if this relationship is causal, the proportion of these injuries attributable to this risk factor in the population was 32% and 43%. Given that there are simple and effective interventions available for both maternal depression [42], [43] and hazardous alcohol use [44] our findings highlight further opportunities for childhood injury prevention.

### Strengths and Limitations of the Study

This is the first and largest study of risk factors for different injury types in pre-school children using population-based nationally representative primary care data in the UK. Since GPs hold information about all aspects of medical care for a patient registered with them, our data are therefore broadly representative of the overall population burden for these injuries rather than the highly selected injuries that present to secondary care. In addition, for some injuries such as fractures, ascertainment is likely to be virtually complete as the vast majority will be medically attended. Whilst we acknowledge the possibility of some incomplete recording of hospital admissions or deaths resulting from accidents, it is likely that such important events will be communicated to the GP and recorded in a child’s record. Furthermore, serious injuries that lead to death represent a very small proportion of overall childhood injuries.

In comparison to most existing studies of injury, a main advantage is that our data were collected prospectively by general practitioners in the clinical setting thus avoiding social desirability and response biases associated with questionnaire-based studies. By virtue of using these routinely collected data on a large population, missing data on potential risk factors were inevitable. Data on Townsend Index were missing for 8% of the sample, however, by including this as a separate category in our analyses, odds ratios for injury showed that this was highly likely to be missing at random. Whilst other studies have shown that overall, recording of information about alcohol consumption behaviour is relatively incomplete in primary care [45] we used records specifically referring to hazardous or harmful alcohol use or alcohol-related morbidity as it is less likely that this would go un-recorded in general practice. Our prevalence falls within population estimates of hazardous or harmful alcohol consumption [46]. In using this method we are not able to assess whether there are also increased risks of injury in households with moderate or less hazardous alcohol consumption behaviours. A further limitation of routine health care data such as that used in our study is that limited information about wider determinants of health such as parental education, residence in temporary accommodation or access to childcare services will be available.

### Comparisons with other Studies

The demographic risk factors we identified for childhood injury, in particular child age, maternal age and birth order were very consistent with those from other studies using different data sources despite their limitations [11], [12], [15], [16], [32], [37]. Few previous studies report fracture as an injury outcome but our findings with respect to child age are consistent with those that do, such as Flavin et al. [38]. Our findings with respect to child sex are consistent with studies of thermal injuries and fractures [19], [32], [47]–[49]. We did not find an association between sex and poisoning, which is consistent with data reported by Beautrais et al. [20] and with the poisoning incidence statistics reported by the World Health Organisation, which showed only a small (0.1/100,000) difference in poisoning rates in Europe between boys and girls [2].

Our finding of a marked effect of increasing deprivation on thermal injury and poisoning risks are also consistent with previous studies using secondary care data [9], [50]. However we found a lack of socioeconomic gradient in fracture risk. Whilst this is consistent with one study [50] it differs from those finding increasing hospital admission rates for long bone fractures with increasing deprivation [9] and higher emergency department attendance rates for fracture among children from the most compared to the least deprived areas [51]. The differences found between our study and others may be because our study included fractures of a range of severities, including those that presented at primary care or emergency departments or that were admitted to hospital.

Apart from specific risk factors that have a direct contribution to injury, there are surprisingly few studies investigating how potential modifiable risk factors of carers may affect the risk of injury in children they are responsible for. In fact, the World Health Organisation’s report on child injury prevention includes only a handful of such articles [2]. We found that children whose mothers had a record of depression in the perinatal period were more likely to have thermal injuries and poisonings up to the age of 5 years and that 17% and 33% of their injuries were attributable to this depression. This is consistent with the scant literature on this subject comprising a 1978 study assessing psychiatric disorder of mothers and children’s accidents more generally [22], two studies of parentally reported injuries from the Avon Longitudinal Study of Pregnancy and Childhood [11], [19] and an Australian study from 1981[20].

Despite increasing concerns about alcohol consumption amongst young men and women in the UK [52] few studies have explored the potential harm to children related to adult alcohol misuse. Evidence from questionnaire-based studies has suggested that parental alcohol consumption may be directly related to risk of childhood injury [25], [26], [28] and our data have shown that adults’ hazardous or harmful alcohol consumption has an important contribution to the burden of childhood thermal injury and poisonings on a population level, with 32% and 43% of these injuries being attributable to household alcohol misuse.

### Implications for Injury Prevention

Injury prevention should include specific injury prevention programmes as well as earlier reduction of modifiable risk factors that put children at risk. NICE guidance recommends that home safety advice is provided by primary care practitioners in the healthy child programme, on home visits and following injuries and that referrals for home safety assessments and safety equipment are made for those at greatest risk [8]. The results of our study show that GPs could run specific searches in the practice, based on the risk factors that we have identified and then share this information with other agencies to inform the targeting of these home safety assessments. In addition, because young children and their parents present frequently to primary care our work has demonstrated that data available within consultations to general practitioners, practice nurses and health visitors could be used for injury prevention opportunistically at such contacts [53]. Our findings suggest that a considerable proportion of thermal injuries and poisonings could be prevented if maternal depression and hazardous or harmful alcohol consumption were prevented or successfully treated and we suggest that policies on education, training and employment could modify communities’ socioeconomic deprivation which is another contributor to injury risk.
